# Developing a dataset of real projects for portfolio, program and project control management research

**DOI:** 10.1016/j.dib.2020.106659

**Published:** 2020-12-17

**Authors:** Brett Thiele, Michael Ryan, Alireza Abbasi

**Affiliations:** a School of Engineering and Information Technology, University of New South Wales, Canberra Australia; b Capability Systems Centre, University of New South Wales, Canberra Australia

**Keywords:** Project management, Portfolio management, Real project data, First principles cost estimation, Bottom-up cost estimation, Resource constrained project data

## Abstract

This article introduces a dataset comprising a portfolio of six projects each having a baseline consisting of a budget derived from a standardized first-principles, bottom-up estimation technique. The projects utilize a homogeneous set of resources, both consumable and non-consumable, which are inter-related in a highly complex, multi-dimensional manner with appropriate correlation between quantity, productivity rates and cost rates. The dataset also includes detailed time-phased actual costs and progress over the life of each project as well as the time-phased values of revenue claimed for each project. The collection and attribution of 12,139 actual costs and the measurement of progress over a period of just under 109 consecutive weeks is consistent and standardized across all six projects providing a unique differentiator to other datasets. The data is valid for research in portfolio, program, or project (PPP) management or control of multiple resource-constrained projects, where projects are managed collectively, generally in a standardized manner to support a strategic business aim.

## Specifications Table

**Table:** 

Subject	Engineering		
Specific subject area	Project, Programme and Portfolio (PPP) cost and schedule management and control.		
Type of data	Linked spreadsheets tables		
How data were acquired	Data was collected from a civil construction company undertaking projects concurrently over an extended and continuous time period. The data collected is owned by the lead author – Brett Thiele who was the Director of the company engaged as the Principal Contractor and acted as the contractor's representative for each project Budget, Cost and Productivity Data: Permission to publish all budget, cost and productivity related data has been given by Brett Thiele. To protect privacy of individuals, names have been removed and replaced with role descriptions such as labor, project supervision, project management and project administration. Revenue Data: Revenue data is the original awarded value and the final approved value after approved scope changes for each contract. As all contracts within this data set were completed for Local Queensland Governments, both the original and final contract values for each project are a matter of public record and are available via an information request to each Local Council. No permission is required to re-publish this data.		
Data format	Raw and filtered		
Parameters for data collection	The projects within the portfolio were completed between 2011 and 2012 with projects ranging between $1M (AUD) and $3.6M (AUD) in value and 4 – 12 months in duration. All work within the projects was completed through self-performance, that is, other than specialized activities the risks associated with resource availability, resource rates and productivity were borne by and managed by the contractor.All projects in the data set were completed on either a fixed lump sum or fixed schedule of rates basis without incentive schemes for an early finish, but damages payable to the client for late finishes. The criteria used for inclusion into the portfolio data set are the projects must: have commenced no earlier than 2011; be completed; have a minimum tender value of $400,000 have an initial planned duration of at least three months and be completed concurrently as part of the portfolio.These criteria led to the acceptance of six projects into the data set with the first project commencing in January 2011 and the final being completed in October 2012. The data has been collected over a continuous period of just under 109 weeks, with the six projects representing the following: 1. Tendered value of $9.402M. 2. A final revenue value of $11,409M. 3. Original combined budget of $9,878M. 4. Final total actual cost of $9,402M. 5. A total of 12,139 individual cost records. 6. A combined planned duration of 1,480 project days. 7. A total combined actual duration of 1,480 project days. Data has been validated at the time of collection monthly. The total resource usage across the portfolio was reconciled as part of the payment of individuals’ wages, payments for resources consumed and sub-contract services monthly. Any discrepancies between recorded resource usage across the portfolio and invoiced amounts were resolved within the monthly period and records adjusted accordingly to reflect true resource usage. This was supported by monthly cash flow and bank reconciliations.12 additional projects exist within the portfolio with associated data; however, these projects are yet to be formatted. As these are completed, the dataset will be augmented until all 18 projects are available.		
Description of data collection	Data was collected from a civil construction company undertaking projects concurrently over an extended and continuous time period. There are three main types of data collected, each of which is described below: Budget Data: Budget data was completed as part of the tendering process for each project and the pricing of ordered scope changes as the project progressed. The baseline budget for each project is produced using a standardized, repeatable bottom up, first principles process utilizing the Client's WBS. Budget costs are derived from the estimated quantity of consumable and non-consumable resources required to complete each task. The budget durations of tasks are determined from the estimated productivity of key non-consumable resources, which are appropriately correlated between tasks to ensure validity of future model runs. The overall budget project duration is determined from the network logic of all tasks, and time-based overheads are determined based on the overall project duration. This process formed the basis upon which tendered prices were submitted to clients. The commercial software *Expert Estimation 2014* was used to develop these budgets and *MS Project* was used to develop project schedules. Cost and Productivity Data: Actual resource use, materials received on site and productivity was recorded daily using a standardized time sheet and site diary with all resource usage and productivity allocated to relevant Portfolio WBS items. Once received at the contractor's head office resources and materials were accrued against the documented Portfolio WBS items within the company project control system, with actual costs determined from agreed cost rates for resources and materials. The commercial project control software *Expert Project* was used for this task. Further information regarding *Expert Estimation 2014* and *Expert Project* software is available at https://pronamics.com.au/. Revenue Data: Revenue data is the original awarded value and the final approved value after approved scope changes for each contract. As all contracts within this data set were completed for Local Queensland Governments, both the original and final contract values for each project are a matter of public record and are available via an information request to each Local Council. No permission is required to re-publish this data.		
Data source location	The geographical locations where each project within the data set was completed is provided below. The co-ordinates provided are approximately the centre of each project site.		
	Project Name and Location	Latitude:	Longitude:
	P01 Airport Car Park. Whitsunday Coast Airport, Lascelles Avenue Gunyarra QLD Australia 4800	20°29′19.22"S	148°33′7.34"E
	P02 Landfill Cell Construction. Kirknie Landfill facility, Home Hill – Kirknie Road, Ayr QLD Australia 4807	19°44′13.63"S	147°17′35.89"E
	P03 Regional Bypass Road. Dysart QLD Australia 4745	22°35′22.24"S	148°21′34.50"E
	P04 Regional Arterial Pavement Repairs. Dysart QLD Australia 4757	22°32′6.99"S	148°18′10.61"E
	P05 Semi-Urban Road Reconstruction. Proserpine QLD Australia 4800 • Nicol Street • Wright Road	20°24′15.44"S 20°21′38.00"S	148°34′25.52"E 148°34′53.39"E
	P06 Urban Roads Reconstruction. Proserpine QLD Australia 4800 Multiple Streets and Roads: • ANZAC Road • Dodd Street • Sterry Street • Dobbins Lane • Cultural Centre Car Park • Jupp Street • Doherty Street • Faust Street	20°24′14.02"S 20°24′9.27"S 20°24′24.99"S 20°24′9.43"S 20°24′8.57"S 20°24′9.35"S 20°24′0.37"S 20°24′0.76"S	148°34′16.02"E 148°34′22.44"E 148°35′0.48"E 148°34′54.84"E 148°34′49.29"E 148°34′16.76"E 148°34′20.45"E 148°34′10.94"E
Data accessibility	Data is hosted on a public repository and may be directly downloaded. Repository name: Figshare Data identification number: https://doi.org/10.6084/m9.figshare.12998822.v2 Direct URL to data https://figshare.com/articles/dataset/Project_Portfolio_Dataset/12998822		

## Value of the Data

• The dataset is important as it is the only known data set of completed real projects that have been delivered as a portfolio across a continuous period of time that utilise a homogeneous set of resources, both consumable and non-consumable, which are inter-related in a highly complex, multi-dimensional manner with appropriate correlation between quantity, productivity rates of key resources and cost rates for materials, labor, plant and equipment and sub-contract resources.
• The data is useful across disciplines and is beneficial to researchers in the fields of Project Management, Project Schedule and Cost Control, Earned Value Management, Resource Constrained Schedule Optimisation, Business, Management and Government policy.
• The data can be used to test hypothesis associated with project control, project cost and duration forecasting and to test algorithms for multiple project resource constrained schedule optimisation research.
• The data enables Probabilistic Risk Modelling and Decision outcomes models to be easily established. Real project outcomes can be used as a comparator for modelled outcomes if various portfolio or project management decisions were taken, for example the data can be used to identify the quantum of financial contingency and the levels at which it should be held within portfolio, program and project scenarios.
• As the projects contain a first principles bottom up estimation technique that is fully correlated between all items within the WBS, the data may be useful to inform the suitability or veracity of fictitious data generated by models.
• The data provides actual cash flow outcomes on a n accrued basis and can be used to model business, management or investment decisions or the suitability of Government policies, especially as the pertain to the financial viability of businesses.

## 1 Data Description

The data set consists of six MS Excel workbooks each of which represents a completed project. Each workbook contains eight linked worksheets which fully describe the original project baseline plan and how the project proceeded. A MS Project template for ease of exporting the resultant linked project networks is also included but does not form part of the dataset.

Key project information is contained in Table 1 and a brief description of the scope of works for each project associated with each datafile is described as follows:

P01 Regional Airport Carpark. This project involved the construction of two new carparks and new vehicle access to the front of the terminal at a mid-size regional airport.
P02 Regional Landfill Cell. This project included the construction of a 2 Ha lined landfill cell and leachate collection system in regional Queensland.
P03 – Regional Bypass Road. This project included the widening and strengthening of approximately three kilometers of arterial road to allow bypass of a regional mining town for heavy vehicles.
P04 – Regional Development Road. This project included the pavement rehabilitation and repairs at multiple sites along a 27 km section of a regional arterial road servicing the local mining industry.
P05 – Rehabilitation Two Roads. This project included the rehabilitation of two roads following the 2011 Queensland floods. Works included the installation of large cross and longitudinal drainage, removal and replacement of saturated subgrades, strengthening with an unbound gravel overlay, bitumen seal and asphalt.
P06 – Urban Street Rehab. This project included the reconstruction of multiple urban streets following the 2011 Queensland floods. Works included improvement of cross and longitudinal drainage, removal and replacement of saturated subgrades, replacement of all kerbing, new gravels, bitumen, and asphalt seal.

**Table 1:** Data set summary

Project No.	Project Name	Start	End	Duration (Days)	BAC	AC	Tender Value	Final Value
P01	Regional Airport Car Park	5/05/11	6/11/11	185	$1,965,174	$1,890,995	$1,902,511	$2,280,303
P02	Regional Landfill	10/01/11	12/07/11	183	$1,183,216	$1,088,404	$1,254,712	$1,310,603
P03	Regional Bypass Rd	6/05/11	18/03/12	317	$1,372,473	$1,336,973	$863,858	$1,652,653
P04	Regional Development Road	24/05/11	25/10/11	154	$944,431	$813,335	$1,080,647	$952,647
P05	Rehabilitation – Two Roads	22/08/11	19/05/12	271	$1,160,892	$1,017,113	$1,394,688	$1,355,086
P06	Urban Streets Rehab	11/10/11	15/10/12	370	$3,251,742	$3,138,824	$2,906,024	$3,858,444
				**1,480**	**$9,877,928**	**$9,285,644**	**$9,402,440**	**$11,409,736**

### 1.1 Composition of individual project data

The data is presented in MS Excel Workbook format, chosen due to the ease at which data is exported to other applications or imported as needed. There are six individual files that make up the data set, with each file containing individual worksheets as follows:

### 1.2 Estimate worksheet

The estimate tab contains the model for the PMB for each project. The PMB for each project is produced using a bottom up, first principles process. Costs are derived based on the quantity of consumable and non-consumable resources whilst the duration of each task is based on the productivity of non-consumable resources to complete a given quantum of work. The duration of the project is then based on the network logic within the Program Links tab. Time based overheads are then determined from the overall project duration. Within each item. the resources needed to complete the task and their associated productivities are correlated or linked to ensure the validity of model runs if sensitivity analysis is undertaken. For example, if an excavator is loading three trucks in a cycle, the overall time required by the excavator and trucks are linked. If a sensitivity analysis is undertaken, the model will not allow the excavator to speed up, whilst the trucks slow down and vice-versa.

The process of building up the PMB is repeatable across the portfolio, and as the project progresses updates to the PMB are easily made to accommodate scope change and provide actual outcomes at the highest project level for ease of comparison.

The estimate contains a user defined WBS number in column A for items and sub-items within it. This is purely for the purpose of assisting with the look up functions associated with sensitivity analysis. The client's WBS descriptors are contained in column B and C respectively. The estimate contains cells which are coloured green. Each of these cells is a variable which can be changed for the purpose of sensitivity analysis. The costs of resources, both consumable and non-consumable are altered in the resources tab.

### 1.3 Resources worksheet

The resources worksheet contains all consumable and non-consumable resources used to build up the baseline. The resources are separated into four types – labor, plant and sub-contractor which are all non-consumable resources and materials which are consumable resources. The UOM and cost rate for each resource is provided here and can be changed globally. The estimate worksheet utilizes a lookup function to insert the rate of the resource. For sensitivity analysis, rates can be modelled with discrete outcomes directed back to the rate cells to inform individual model runs.

### 1.4 Model inputs worksheet

As previously noted, the estimate contains green cells, each of which is a variable that can be altered for the purpose of sensitivity analysis. The model inputs worksheet contains all variables, with UOM noted and the type of the variable, either quantity or productivity based. Again, for sensitivity analysis quantities and productivity rates can be modelled with discrete outcomes directed back to the model input cells to inform individual model runs.

### 1.5 Non-workdays worksheet

The non-workdays worksheet contains the non-workday calendar in the locality the projects were completed for the portfolio plus an additional two years from 2011 – 2015 to assist in modelling, the start date for project and the base working hours per day. It is provided to ensure the networks produced are aligned with the timespans of actual delivery and the costs accrued have meaning. The working hours per day is utilised within the estimate to determine the duration of each task and can be changed globally. The portfolio calendar is incorporated into the MS Project template provided with the dataset.

### 1.6 Program links worksheet

The program links worksheet contains the network logic required to construct the project program, together with the actual progress against each project activity over the life of the project. Copying column A to E into the provided MS Project template and auto-generating the schedule produces a fully linked project network complete with durations and direct cost budgets for each task.

### 1.7 Budget and revenue worksheet

The budget and revenue worksheet contains the actual direct cost budget and contract rates, totals and agreed progress for the project based on the client's WBS and includes all project variations. For the purpose of generating progress claims, revenue and reporting to the client, the progress of each WBS item was measured and agreed, generally on a monthly basis, which then formed the basis of payment claims and updates to the project program. This worksheet contains the agreed measurement and revenue paid for the project on a time phased basis.

### 1.8 Portfolio WBS worksheet

The Portfolio WBS worksheet dissects the project into the Portfolio WBS (PWBS) format and describes the project in the following manner: The baseline is described via the planned value (PV) over time of the project, with the PV of each PWBS item being the planned quantity (PQ) of the work at the completion of each month multiplied by the PWBS item budget.

The actual progress of the project is shown as the actual quantity (AQ) completed for each PWBS item at the end of each month, with the earned value (EV) being the AQ multiplied by the PWBS item rate. Actual costs (AC) for each PWBS item are summarized on a cumulative basis at the end of each month.

### 1.9 Actual costs worksheet

The actual costs worksheet is a full account of all costs associated with the project on the date to which they were accrued. They are summarized by PWBS item in date order with the resource, quantity, rates, and totals shown.

Actual resource usage and materials received on site were recorded daily on a standardized time sheet and allocated to relevant PWBS items. Once received in the head office, usually within 24 hours, resources and materials were accrued against each PWBS item within the company finance and project control system, with actual costs calculated based on the contract or agreed cost rates for resources and materials.

This standardized method enabled a simple manner of data collection from the field and provided a sound feedback mechanism for the initial first-principles cost-estimation process as project monitoring and control occurred in the same manner used to establish the baseline.

To protect privacy of individuals, names have been removed and replaced with descriptions such as labor, project supervision, project management and project administration. A time phased summary of the AC by PWBS items is also provided.

### 1.10 Post action

The data set is presented as post action that is as projects progressed, necessary corrective actions were taken to ensure a minimization of actual costs and maximization of production and profit. This is a valid position, supported by Vanhoucke, et al. [1] who note the main reason project data is collected in the first instance is to support the decision making of corrective actions and not for the benefit of researchers.

### 1.11 Data-scrubbing

In the compilation of this data set each of the 12,139 individual cost entries has been reviewed for accuracy and validity to ensure that they have been allocated against the appropriate PWBS item. Any discrepancies identified have been rectified, such as gravel supply being incorrectly allocated to concrete supply, or a sewer installation sub-contractor being allocated to a water main installation sub-contractor for example. It is acknowledged that because some data entries required re-allocation there is the possibility that others have been missed, potentially leaving ‘noise’ in the data set and it is therefore not perfect, however it is believed to be as accurate as it can be.

### 1.12 Work Breakdown structures (WBS) client based and standardized portfolio WBS

The above description notes the use of client based WBS and a standardized PWBS. The following is a brief description of why this was done and why it is important to the dataset.

A WBS is a decomposition of a project into smaller discrete tasks that collectively, fully describe the work to be completed to improve reporting and control at the project level. Readers are directed to PMI [2] which fully describes the WBS, the various typical formats a WBS might take, the characteristics of a high-quality WBS and what a high-quality WBS should be capable of achieving within the project, program or portfolio environment. GAO [3] also provides further information.

The bottom-up first principles estimates used to determine the baseline budgets for all projects within the dataset utilize the client's WBS as the starting point as it is normal for a client to provide a project WBS to a contractor for them to provide price and program against. The client's WBS is generally structured to assist them monitor and report on progress either internally or externally.

WBS structures provided by the client very rarely follow the decomposition of a project that best suits project control and reporting within a contractor's organization, nor do they generally make direct reference to cost items often referred to as overhead costs, such as a contractor's project management, project supervision, payment of insurances, levies or permits, the hire of site facilities or the provision of staff vehicles and accommodation - just to name a few.

As each client's WBS is generally different, the use of the client's WBS for contractor's cost and schedule control requires bespoke resource and cost accrual methods for each project. For this reason, this dataset utilizes a standardized Portfolio WBS (PWBS) has been developed for this data set, based on typical standardized, repeated tasks across the portfolio, enabling each project to be described in a standard manner simplifying reporting and control. The PWBS is broken into three main categories of Materials, Production and Overheads.

Materials. All materials or consumable resources have their own PWBS code with a unit of measure (UOM) commensurate with how the resource is purchased, and without distinction of which tasks the material is used. This is valid as the cost of purchase and delivery of consumable resources to a project site is independent of the effort required to incorporate them into the work.

Production. Production PWBS codes are effort driven, are based on the use of non-consumable resources and do not include the supply of materials. The UOM associated with the PWBS codes enable direct measurement of productivity from the field without the need for detailed survey or complex measurements.

Overheads. Overhead items are those tasks associated with a project that do not generally form part of the direct costs associated with a specific client WBS. Upon submission by a contractor of a client's WBS, overhead costs are generally spread or apportioned across all WBS items, making tracking and attribution of them using the client WBS a complex task requiring a disproportionate effort from the project staff.

**Fig. 1:**
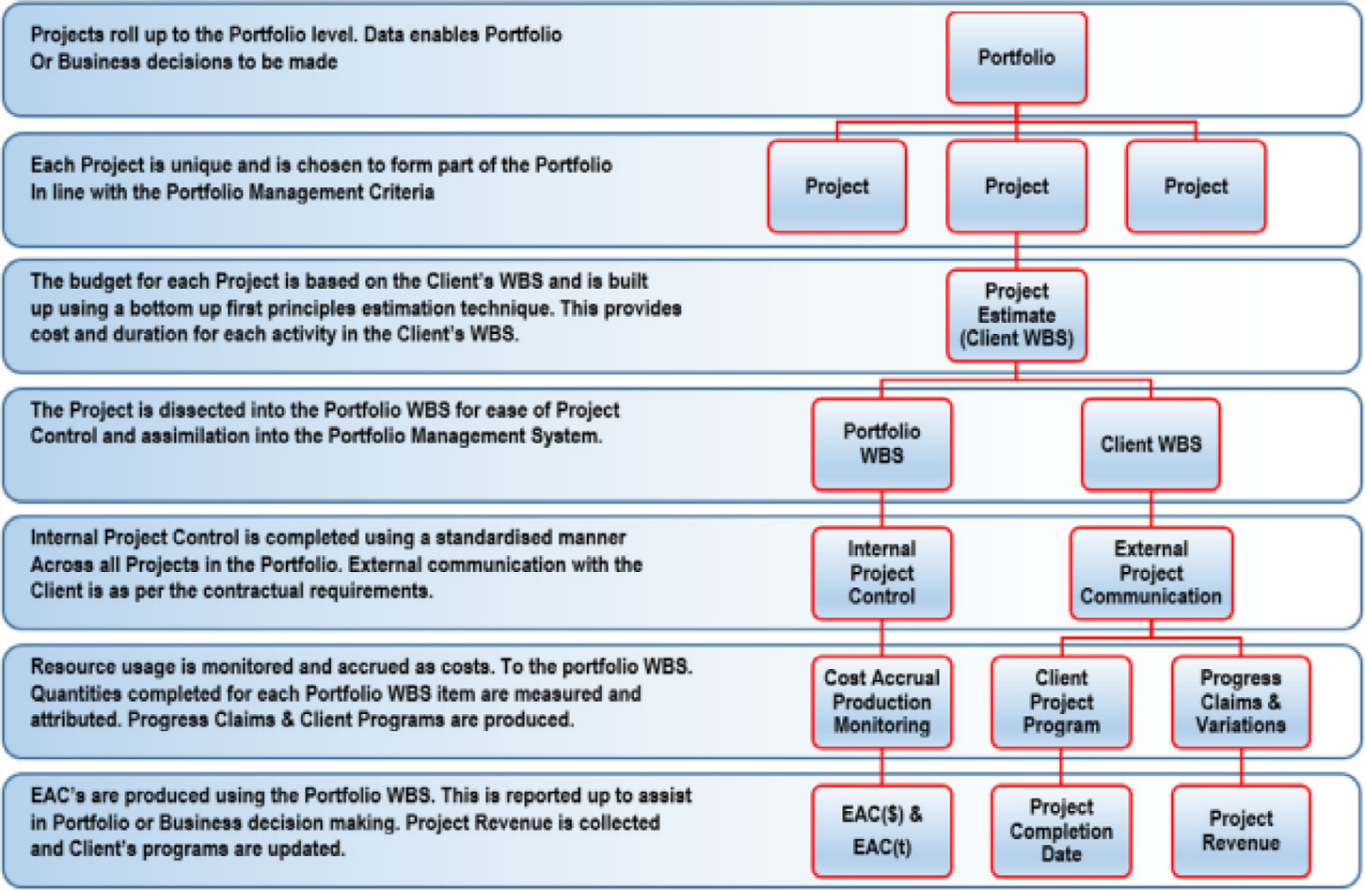
Relationship and usage of PWBS and client's WBS.

**Fig. 2:**
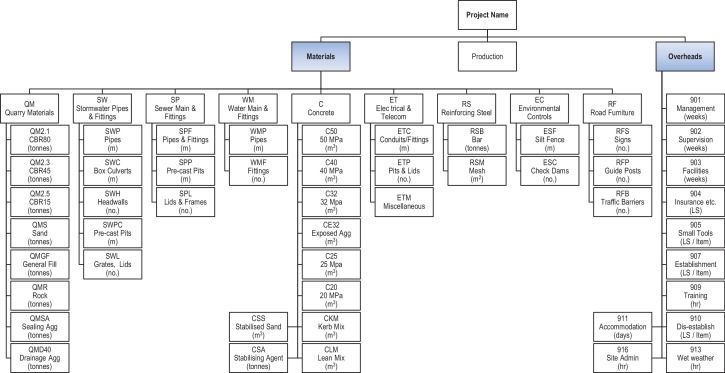
PWBS (Materials and Overheads).

**Fig. 3:**
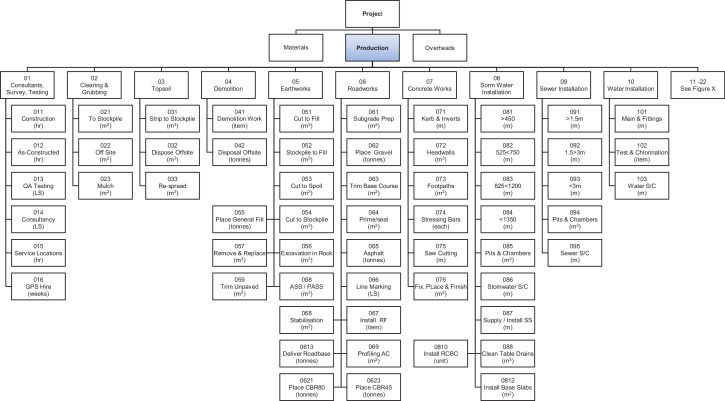
PWBS (Production Part One).

**Fig. 4:**
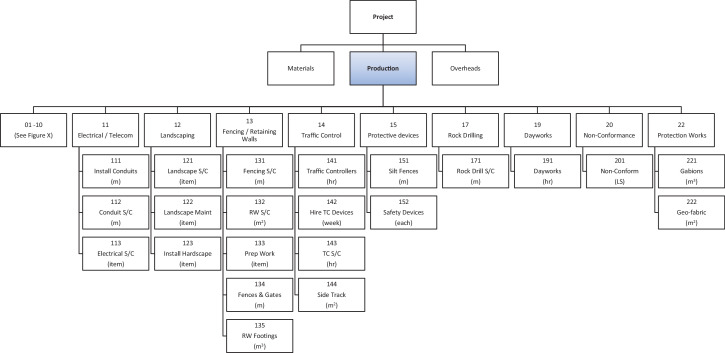
PWBS (Production Part Two).

The relationship between the clients WBS and the PWBS is shown in Fig. 1 with the PWBS extracted from the project estimate to enable standardized project cost and schedule control across multiple projects, whilst the Client's WBS is used to communicate the project position to the client in the manner to which they wish it to be reported. A description of the internal (contractor) and external (client) uses at each step of the WBS and the PWBS used in the dataset is included. Fig. 2 Fig. 3 Fig. 4 show the PWBS decomposition to the lowest task levels for the three main categories of materials, production and overheads.

The PWBS maintains the characteristics nominated by PMI [2] and follows the example given in GAO [3]. It fully describes the work at the lowest level without being onerous and it does not rely on or follow project network logic. The underlying principle of the PWBS is twofold, firstly to enable a project team from the outset to easily quantify the amount of consumable resources needed and the tasks required to complete the works. The second is to provide a simple method of tracking progress against the PWBS codes that can be reported daily from site-based personnel. By doing this in a standard manner across the portfolio, individuals can transfer between projects without the need for re-training in administrative functions, and methods for determining percentages complete of works and estimates of cost and time at completion across all projects are standardized.

## 2 Experimental Design, Materials and Methods

An extensive review of the literature did not identify any definitive characteristics of a good data set for research within portfolio, program, or project management; therefore, the following eight characteristics were established and used.

Standardized baseline determination for all projects. The baselines (budget and durations) for each project were determined in a standardized manner. This enabled replication across the portfolio and changes to be completed globally simplifying sensitivity or ‘what if’ scenario analysis. To complete this, the key aspects of project control – quantum of work, resource costs and availability and productivity must be easily altered for modelling purposes.

Standardized data collection techniques. As each project progressed, the data was collected in a standardized manner. This method is easily repeatable and enabled all data collected across the portfolio to be treated in the same manner and allows additional data to be added to the set if additional works are ordered within a defined framework.

Data attribution. Each individual data record is attributed to a defined, quantifiable, and measurable outcome at the lowest possible level with each project, such that outcomes are discrete and definable, yet the data collection is not onerous. This attribution gives the data meaning and purpose, so it is possibly the most important characteristic of the data set. For example, the production or collection of cost-related data against a discrete physical quantity of measurable work allows actual unit rates to be established. If cost data is not attributed to discrete quantities or the outcome attributed is at too high a level, the establishment of unit rates becomes problematic which in turn introduces estimation errors and reduces forecast accuracy.

Collected data must equate to the entire project. The data presented in this dataset represents the entirety of each project within the portfolio. This is important as a portfolio looks holistically at the overall performance of the projects within it, the data collected must be truly representative of the entire project and not just a part or component.

Portfolio data is continuous across an extended time period. As a portfolio looks holistically across the multiple constituent projects, the data production or collection process must be continuous across an extended period to ensure that there are no gaps. The period should extend such that the quantity of records is sufficient for the data set to be considered statistically significant.

Consistent time intervals for data summarization across all projects. The time interval or reporting date at which data is summarized as the projects are planned or progress is consistent. This allows summation at the program or portfolio level to occur with reference to a standard data date. For individual projects, additional data dates can be introduced in between the portfolio data date for the purposes of control, however the portfolio data date should always be honored.

Data collected is easily compared to project baselines. This may appear self-evident; however, the method of establishing the initial project baselines and the method of tracking and collecting data can be very dis-similar, depending upon the purpose, use or target audience of the data. The method used to establish each project baseline and dissect the budget into the PWBS in this dataset is standardized and directly relates to the method used to attribute collected data and hence forecast Estimates at Completion for both cost and time.

Portfolio data includes revenue information. This dataset includes the time-phased revenue generated by each project in the portfolio, and includes the approved amounts claimed and paid by clients. Revenue is an important factor in portfolio research as the structure and spread of costs and margin across each project within the portfolio directly influences the cashflow, and therefore health of the portfolio itself.

## Ethics Statement

The work does not include the use of human subjects, animal experiments or data collected from social media platforms. Cost records have had the identity of individuals deleted, whilst legal entities (companies) remain identifiable within the dataset.

## Credit Author Statement

**Brett Thiele:** collected the original data, formatted if for consistency and is the principal author of this article. **Michael Ryan:** contributed to the design of the data set and this article and is a secondary author. **Alireza Abbasi:** contributed to the design of the data set and this article and is a secondary author.

## Declaration of Competing Interest

The authors declare that they have no known competing financial interests or personal relationships which have or could be perceived to have influenced the work reported in this article.
